# Role of Kupffer Cells in Thioacetamide-Induced Cell Cycle Dysfunction

**DOI:** 10.3390/molecules16108319

**Published:** 2011-09-29

**Authors:** Mirandeli Bautista, David Andres, María Cascales, José A. Morales-González, María Isabel Sánchez-Reus, Eduardo Madrigal-Santillán, Carmen Valadez-Vega, Tomas Fregoso-Aguilar, Jorge Alberto Mendoza-Pérez, José Gutiérrez-Salinas, Jaime Esquivel-Soto

**Affiliations:** 1 Instituto de Ciencias de la Salud, Universidad Autónoma del Estado de Hidalgo, Ex-Hacienda de la Concepción, Tilcuautla, 42080 Pachuca de Soto, Hgo, Mexico; Email: jmorales101@yahoo.com.mx (J.A.M.-G.); eomsmx@yahoo.com.mx (E.M.-S.); maryna_valadez@hotmail.com (C.V.-V.); 2 Instituto de Bioquímica (CSIC-UCM), Facultad de Farmacia, Ciudad Universitaria, Plaza de Ramón y Cajal S/N, 28040 Madrid, Spain; Email: cascales@farm.ucm.es (M.C.); mireus@farm.ucm.es (M.I.S.-R.); 3 Escuela Nacional de Ciencias Biológicas, Instituto Politécnico Nacional, México, D.F., 07700, Mexico; Email: fisiobiologo@hotmail.com (T.F.-A.); jorgemendozaperez@yahoo.com (J.A.M.-P.); 4 Laboratorio de Bioquímica y Medicina Experimental, División de Investigación Biomédica, Centro Médico Nacional “20 de Noviembre”, ISSSTE, México, D.F., 03229, Mexico; Email: quauhtlicutli@yahoo.com (J.G.-S.); 5 Facultad de Odontologia, Circuito Escolar S/N, Ciudad Universitaria (UNAM), México, D.F., 04510, Mexico; Email: jaime_esquivel2003@hotmail.com (J.E.-S.)

**Keywords:** gadolinium chloride, kupffer cells, thioacetamide hepatotoxicity, cell cycle, cyclins

## Abstract

It is well known that gadolinium chloride (GD) attenuates drug-induced hepatotoxicity by selectively inactivating Kupffer cells. In the present study the effect of GD in reference to cell cycle and postnecrotic liver regeneration induced by thioacetamide (TA) in rats was studied. Two months male rats, intraveously pretreated with a single dose of GD (0.1 mmol/Kg), were intraperitoneally injected with TA (6.6 mmol/Kg). Samples of blood and liver were obtained from rats at 0, 12, 24, 48, 72 and 96 h following TA intoxication. Parameters related to liver damage were determined in blood. In order to evaluate the mechanisms involved in the post-necrotic regenerative state, the levels of cyclin D and cyclin E as well as protein p27 and Proliferating Cell Nuclear Antigen (PCNA) were determined in liver extracts because of their roles in the control of cell cycle check-points. The results showed that GD significantly reduced the extent of necrosis. Noticeable changes were detected in the levels of cyclin D1, cyclin E, p27 and PCNA when compared to those induced by thioacetamide. Thus GD pre-treatment reduced TA-induced liver injury and accelerated the postnecrotic liver regeneration. These results demonstrate that Kupffer cells are involved in TA-induced liver and also in the postnecrotic proliferative liver states.

## 1. Introduction

Kupffer cell function plays an important role in the pathogenesis induced by hepatotoxic compounds [1]. Kupffer cells are the macrophages residing in the sinusoids of the liver. This makes Kupffer cells the first macrophage population to come in contact with noxious materials (bacteria, virus, tumor cells or drugs). Thus, blocking of Kupffer cells function significantly reduces the hepatotoxicity.

Gadolinium chloride (GD) is a selective Kupffer cell toxicant that completely eliminates large Kupffer cells from the liver and has been extensively used in mechanistic studies of hepatotoxic processes [2]. Kupffer cells exhibit intraacinar heterogeneity, since those located in the periportal area are larger and exhibit higher phagocytic activity compared with those located in the perivenous area [3]. It is well known that the function of these cells (release of cytokines and proteases, superoxide anion production, *etc.*) plays an important role in the pathogenesis induced by hepatotoxic compounds [1,4]. GD most likely is protective because it prevents the release of inflammatory cytokines and toxic oxygen radicals produced by activated Kupffer cells [5,6].

The acute liver injury induced by a necrogenic dose of thioacetamide (TA), a potent hepatotoxic agent, is characterized by a severe perivenous necrosis [7,8]. The necrosis develops as a consequence of the biotransformation of TA through the microsomal flavin-dependent monooxygenase. The reactive metabolites responsible for TA hepatotoxicity are the radicals derived from thioacetamide-*S*-oxide and the reactive oxygen species derived as subproducts in the process of microsomal TA oxidation; both of wich can deplete reduced glutathione leading to oxidative stress [9,10].

In animal cells the cell proliferation are regulated primarily in G1 phase. At the end of this phase, the so-called restriction point, mitogenic signals are integrated and the cells proliferating. The restriction point defines a process of “no return” so that once cells pass it; undertake to carry out a full cell cycle even if the mitogenic signal disappears. The progression of cells along the cycle is controlled by cyclin-dependent protein kinases (CDKs), activated as a result of their association with some regulatory proteins called cyclins.

The cyclins are synthesized in a cell cycle phase, activate the corresponding CDK and subsequently are degraded by the proteasome. Thus, in G1 CDK4 and CDK6 complexes are activated in combination with D cyclins, and CDK2 with cyclin E [11,12]. Cyclin A functions later in S phase [13] as well as on the transition G2/M complexed with CDK2 and CDK1, respectively. Finally, the activation of CDK1-cyclin B complex is necessary to promote entry into mitosis, which phosphorylates a large number of substrates that determine the G2-metaphase transition [14,15].

In order to deepen our current understanding of these processes and try to clarify the mechanisms responsible for this advancement in the process of liver regeneration observed in rats pretreated with gadolinium [16], we decided to analyze the levels of a number of proteins involved in the control of various strategic points in the cell cycle as are the restriction point and entry into DNA synthesis phase.

As it is generally accepted that the Kupffer cell function is involved in the severity of liver damage induced by drugs, and that GD induces a selective blockade of Kupffer cell function when administered intravenously, the purpose of the present study was to elucidate the role of Kupffer cells in the regeneration after liver injury, blocking specifically Kupffer cells function by GD. The effect of GD was assayed on an experimental model of liver injury induced by a single necrogenic dose of TA which results in necrosis in the perivenous acinar area. Groups of rats were pre-treated or not intravenously with GD 24 h before TA.

The proliferative post-necrotic response was assayed by evaluating levels of cyclin D1, cyclin E, p27 and Proliferating Cell Nuclear Antigen (PCNA) because of their roles in the control of cell cycle check-points.

## 2. Results

### 2.1. Effect of GD on Parameters of Liver Necrosis

Liver damage induced by xenobiotics is characterized by the release in serum of hepatic enzymes due to necrosis of hepatocytes. AST and ICDH are two enzymes used as markers of necrosis. AST is randomly distributed in the hepatic acinus, while ICDH is mainly located in the perivenous acinar region, ICDH activity is used as a parameter of hepatocellular damage to measure the severity of centrilobular necrosis *in vivo*. Serum ICDH is the best marker for perivenous necrosis since aspartate and alanine aminotransferases are mainly located in the periportal space [17].

**Figure 1:**
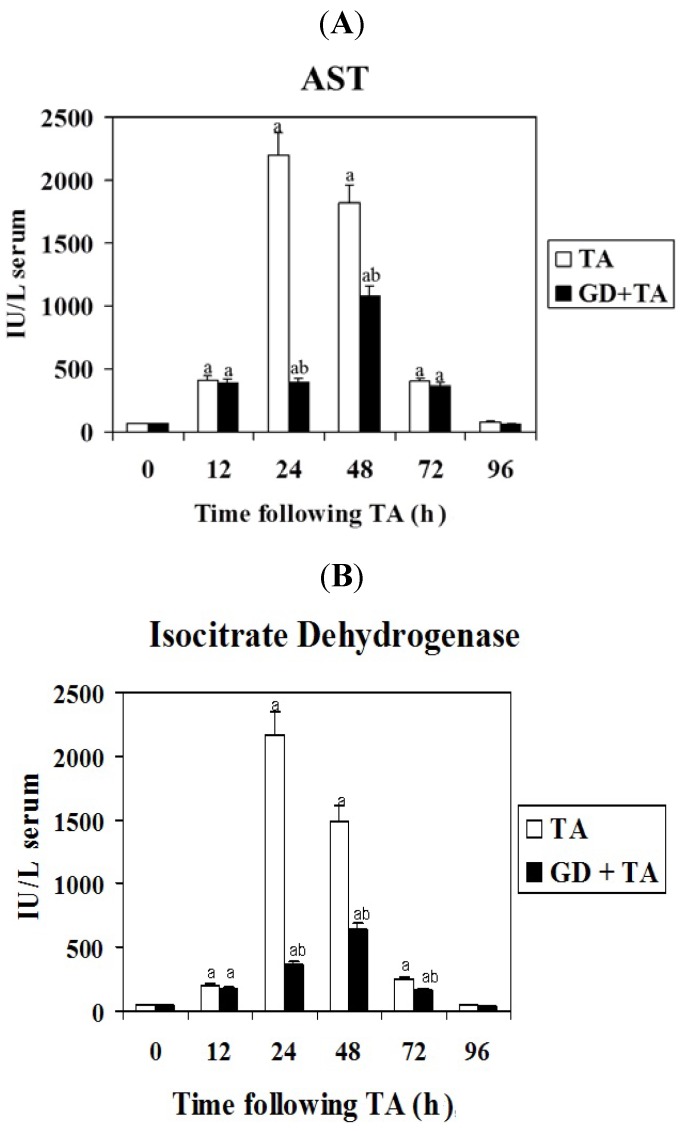
Effect of GD pre-treatment on aspartate aminotransferase (**A**) and isocitrate dehydrogenase (**B**) activities in serum of rats intoxicated with one sublethal dose of thioacetamide. Samples were obtained at 0, 12, 24, 48, 72 and 96 h following thioacetamide (TA). The results, expressed as IU/L of serum, are the mean ± SD of four determinations in duplicate from four rats. Differences against the respective control are expressed as (a) and differences due to GD are expressed as (b), *P* < 0.05.

**Figure 2:**
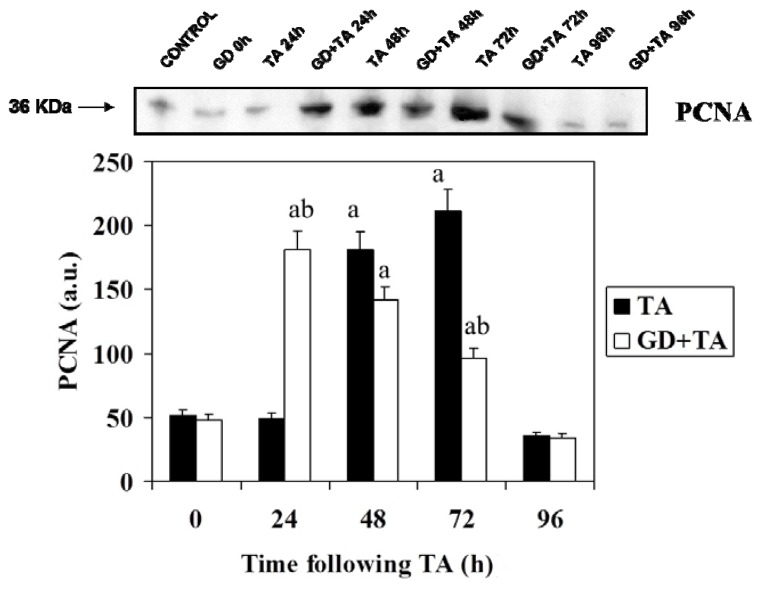
Effect of GD pre-treatment on levels PCNA protein assayed by Western blot in liver homogenates of rats intoxicated with one sublethal dose of thioacetamide. Samples were obtained at 0, 24, 48, 72 and 96 h following thioacetamide (TA). The results, expressed in arbitrary units, are the mean ± SD of four determinations in duplicate from four rats. Differences against the respective control are expressed as (a) and differences due to GD are expressed as (b) p < 0.05.

The increase in both activities was detectable at 12 h of TA administration and reached the maximum at 24 h (Figure 1 and Figure 2). The extent of necrosis induced by TA was detected by a peak of 30 and 45 times the basal values, for AST and ICDH activities respectively. When rats were pretreated with GD the 24 h peak values were reduced to 15% and 16% for AST and ICDH respectively. However, at 48 h of intoxication the difference due to GD were 56% and 43% for these enzyme activities, which indicate that GD delays TA-induced liver injury, since the maximum of necrosis appeared at 48 h of intoxication. No effects were detected on serum activities when GD was administered without TA (data not shown).

### 2.2. Effect of GD Pretreatment on Cyclin D1, Cyclin E, p27 and PCNA Level in Liver of Rats Following the Intoxication of TA

Figure 2 shows the Western blot and the quantification of signals corresponding to PCNA. PCNA is a protein that works as an auxiliary protein of DNA polymerase-δ and enhances DNA replication. The maximum and significant increase can be observed in the level of this protein in rats pretreated with GD at 24 h, while in the group of rats treated with TA, the highest level is reached between 48 and 72 h. In both groups, once the maximum is reached, the levels of PCNA decreased up to 96 h where the values return to basal. These results are in agreement with those obtained by flow cytometry in our previous studies [16].

**Figure 3:**
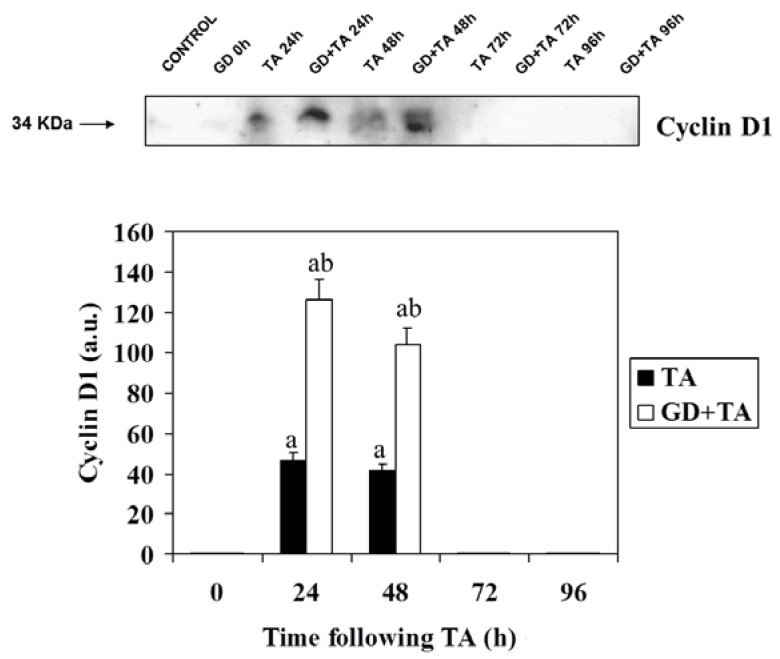
Effect of GD pre-treatment on levels Cyclin D1 protein assayed by Western blot in liver homogenates of rats intoxicated with one sublethal dose of thioacetamide. Samples were obtained at 0, 24, 48, 72 and 96 h following thioacetamide (TA). The results, expressed in arbitrary units, are the mean ± SD of four determinations in duplicate from four rats. Differences against the respective control are expressed as (a) and differences due to GD are expressed as (b) p < 0.05.

Figure 3 shows the immunoblotting of cyclin D1 and its quantification. Cyclin D1 forms complexes with CDK4 and CDK6 that are implicated in the phosphorylation of pRb. Large differences were found in cyclin D1 levels at 24 and 48 h *versus* controls. These data corroborate the increased replication of hepatocytes observed by flow cytometry in the cell cycle [16]. The increase of Cyclin D1 is more much pronounced in rats pretreated with GD, which agree with the data of PCNA and the percentages of S1 population [16].

**Figure 4:**
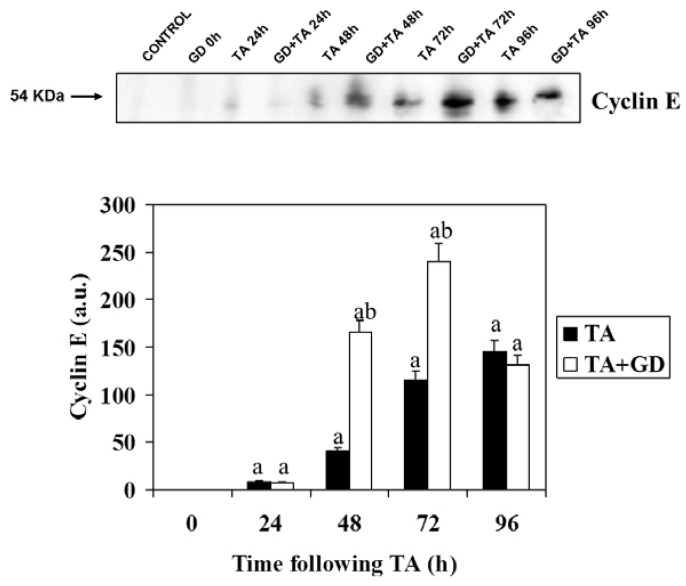
Effect of GD pre-treatment on levels Cyclin E protein assayed by Western blot in liver homogenates of rats intoxicated with one sublethal dose of thioacetamide. Samples were obtained at 0, 24, 48, 72 and 96 h following thioacetamide (TA). The results, expressed in arbitrary units, are the mean ± SD of four determinations in duplicate from four rats. Differences against the respective control are expressed as (a) and differences due to GD are expressed as (b) p < 0.05.

Cyclin E plays a key role in activating the G_1_/S transition. It is periodically expressed at the end of G_1_ and forms complexes preferentially with CDK2. Cyclin E/CDK2 complexes are involved in maintaining the phosphorylation state of pRb. Figure 4 shows a representative Western blot and the quantification of signals corresponding to cyclin E. It can be observed, in both groups of rats (TA, and GD + TA), how the levels of the protein drastically increase from 48 h versus the controls, reaching the highest value at 72 h in rats pretreated with GD, and at 96 h in rats treated with a single dose of TA. We can see again that the increase is more much evident in rats pretreated with GD getting approximately a 400 and 200% of increase versus rats treated with TA at 48 and 72 h, respectively.

**Figure 5:**
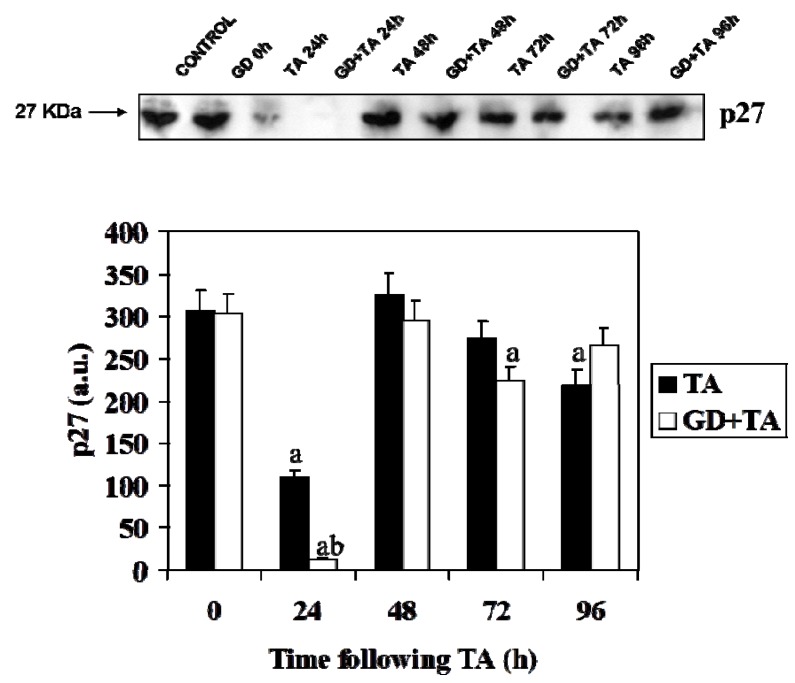
Effect of GD pre-treatment on levels p27 protein assayed by Western blot in liver homogenates of rats intoxicated with one sublethal dose of thioacetamide. Samples were obtained at 0, 24, 48, 72 and 96 h following thioacetamide (TA). The results, expressed in arbitrary units, are the mean ± SD of four determinations in duplicate from four rats. Differences against the respective control are expressed as (a) and differences due to GD are expressed as (b) p < 0.05.

p27 is a cyclin-dependent kinase inhibitor that regulates cell number and size by blocking initiation of a G_1_ buildup by binding to G_1_-specific cyclin-dependent protein kinases. Figure 5 shows the immunoblotting detection and signal quantification of p27. The level of this protein drops drastically at 24 h to restore immediately afterwards to basal values. The decrease of p27 occurs simultaneously to the increase of cyclin D1 and PCNA.

## 3. Discussion

In the present study TA-induced hepatotoxicity was used to investigate the effect of a previously administered single dose of GD on the multistep events involved in liver regeneration. The results obtained in the present paper provide evidence that GD, when administered intravenously prior to TA, significantly enhances the liver regeneration.

Kupffer cells play an important role in the hepatic response to injury; they display upregulated scavenger functions and produce various inflammatory mediators including cytokines and reactive oxygen species [18]. Kupffer cells and infiltrating neutrophils contribute to liver injury in different experimental models of hepatotoxicity [19,20]. In our experiments, GD significantly attenuates liver damage; this attenuation is parallel to Kupffer cell function since the levels of cytokines like TNFα were significantly reduced [16]. Thus, it is clear that the mechanism of this protection seems to result from a diminished generation of ROS and inflammatory and cytotoxic mediators (cytokines and proteases) released from Kupffer cells.

When quiescent cells are stimulated to proliferate, the first cyclin that is synthesized its cyclin D, which is expressed in late G1 phase. Cyclins D (D1, D2, and D3) are an exception to the cyclins, because are expressed as a constant, as long as the growth factor signal remains outside the cell [21]. The loss of cyclin D1, before exceeding the restriction point in G1 prevents cells from entering S phase, but its absence has no effect once turned this point. Therefore, cyclin D1-CDK complexes had to phosphorylate some substrates that are necessary to be modified to exit G1, being the retinoblastoma tumor suppressor protein (Rb) one of their targets. The phosphorylation of Rb, initially triggered by the cyclin D-dependent CDK and then accelerated by the CDK2-cyclin E complexes, causes the release of these E2F, allowing transactivation of these genes [22,23].

For its part, the p27 protein belongs to a family of CDK inhibitors and appears to be directly involved in restriction point control thus in quiescent cells its level is high, while decreases after a cell enter to the cell cycle.

Based on results of other authors, who consider the cyclin D1 and p27 protein mainly responsible for restriction point control of the cell cycle, we decided to determine the hepatic levels of these proteins in groups of rats treated with TA and GD + TA. The results show that the concentration of cyclin D1 experienced a marked increase at 24 and 48 h by effect of TA administration, returning to baseline at 72 h. The same profile was observed in rats pretreated with gadolinium chloride (GD + TA), although it is noteworthy that cyclin D1 levels are achieved in this group two times higher than those observed in rats treated with TA alone. This fact, together with the steepest decline occurring in p27 levels in rats pretreated with gadolinium chloride, explains that the passage through the restriction point and entry into the cell cycle forward and make in a greater number of cells, in the liver of rats who Kupffer cells function was inhibited, as demonstrated by observing the increase in the percentage of cells that are undergoing DNA synthesis determined by flow cytometry [16].

Cyclin E is another protein that plays an important role in activating the G1/S transition and periodically expressed in proliferating cells at the end of G1 phase forming complexes with CDK2 that maintain pRb phosphorylation. It seems that while the D cyclins are integrators of extracellular signals with cell cycle machinery, cyclin E may be crucial for the activation of initiation of DNA replication and hence for entry into S phase [9] The results of our experiments show that increasing the concentration of this cyclin is much higher in rats pretreated with gadolinium chloride, which confirms and supports the data of cyclin D1, p27 and cell cycle, thus advancing regenerative liver cell proliferation of this group of rats.

To complete the study of proteins involved in cell cycle control and proliferation process, we determined the levels of proliferating cell nuclear antigen (PCNA), a key protein in the cell division cycle, which is involved in the process DNA synthesis because it acts as an auxiliary δ DNA polymerase, and relate with DNA repair by joint action with the DNA polymeraseε. In a normal division cycle, the highest expression of PCNA is reached in the transition G1/S and later decrease in G2/M [24].

At present it is considered that PCNA is one of the best markers of cell proliferation [25,26], but cannot be regarded as a marker of S phase by virtue of their lifetime between 8 and 20 hours, which means that their detection may not only affects the cells involved in this phase but also involved in G2 and M [27,28]. This proliferation marker indicates us, as in the case of cyclins D1 and E, that the process of cell proliferation on liver regeneration post necrotic significantly ahead in rats pretreated with the inhibitor of Kupffer cells, as can be seen PCNA increased after 24 h in rats GD + TA, while this increase occurs later, at 48 h in the group of rats treated with TA alone.

Depletion of Kupffer cells with GD seems to increase hepatocyte proliferation and liver regeneration following partial hepatectomy [14,15]; however, the mechanism responsible remains unknown. The hypothesis proposed by other authors who assigned a crucial role in the advancement TNFα and acceleration of cell proliferation, is ruled out in our experimental model [16], since the release of this cytokine decreases significantly by inhibit the function of Kupffer cells, main source of TNFα in the liver.

## 4. Experimental

### 4.1. Reagents

Enzymes were obtained from Boehringer Mannheim (Mannheim, Germany). Substrates and coenzymes were from Sigma (St Louis, MO, USA). Standard analytical grade laboratory reagents were obtained from Merck (Darmstadt, Germany). Antibodies for Western-blot analysis were obtained from Santa Cruz Biotechnology, Inc (California, CA, USA). PVDF membranes and chemiluminescence reagents by Amersham Life Science (Madrid, Spain).

### 4.2. Animals and Treatment

Male adult Wistar rats (2 months old, 200–220 g) were obtained from PANLAB (Barcelona, Spain), and acclimated to our animal room for two weeks before use. Throughout these two weeks rats were supplied with food and water *ad libitum,* exposed to a 12 h light-dark cycle and given intraperitoneally a single necrogenic dose of thioacetamide (6.6 nmol/Kg body weights), freshly dissolved in 0.9% NaCl. The dose of thioacetamide was chosen as the highest dose with survival above 90% [9,10]. GD pre-treatment was performed 24 h before thioacetamide. GD was dissolved in 0.9% NaCl and administered in a tail vein (0.1 mmol/Kg body weight). Untreated animals received 0.5 mL of 0.9% NaCl. Samples of blood and liver were obtained from rats at 0, 12, 24, 48, 72 and 96 h following thioacetamide [29]. Experiments were performed on two different groups: rats treated with a single dose of thioacetamide (TA) and rats pre-treated with GD and treated with a single dose of thioacetamide (GD + TA). Each experiment was performed in duplicate from four different animals and followed the international criteria for the use and care of experimental animals outlined in T*he Guiding Principles in the use of Animals in Toxicology* adopted by the Society of Toxicology in 1989.

### 4.3. Processing of the Samples

In order to clarify the sequential changes during the different stages of liver injury and the post-necrotic regenerative response, samples were obtained from control and at 12, 24, 48, 72,and 96 h of TA intoxication in both GD pretreated or non-pretreated animals. Rats were sacrificed by cervical dislocation and samples of liver were obtained and processed as previously described [30]. Blood was collected from hearts and kept at 4 °C for 24 h, centrifuged at 3,000 rpm for 15 min, and serum was obtained as the supernatant.

### 4.4. Determination of Enzymes and Metabolites

Enzymatic determinations were carried out in serum in optimal conditions of temperature and substrate and cofactor concentrations. Aspartate aminotransferase (AST) and isocitrate dehydrogenase (ICDH) activities were determined in serum. AST (EC 2.6.2.1) activity was assayed following the method of Rej and Horder [31]. ICDH (EC 1.1.1.39) was determined as described previously [32].

### 4.5. Immunoblotting for Detection of Cyclin D1, Cyclin E, PCNA and p27

The proliferative post-necrotic response was assayed by evaluating levels of cyclin D1, cyclin E, p27 and Proliferating Cell Nuclear Antigen (PCNA) because of their roles in the control of cell cycle check-points. Total protein extract was obtained as follows: liver tissue (100 mg) was homogenized in lysis buffer (1 mL) containing Tris-HCl (10 mM), NaCl (200 mM), EGTA (1 mM), Nonidet P-40 (0.5%), β-mercaptoethanol (5 mM), glycerol (5%), Cl_2_Mg (1 mM) and the protease inhibitors phenylmethylsulfonyl fluoride (PMSF, 0.5 mM), aprotinin (40 μg/mL) and leupeptin (4 μg/mL).

Protein concentrations were determined by the method of Bradford [33]. Total protein extracts were boiled in equal volumes of loading buffer (125 mM Tris-HCl, pH 6.8, 4% SDS, 20% glycerol and 10% 2-mercaptoethanol). Protein levels were then analysed by Western blot. Aliquots containing equal amounts of protein (20 μg) were loaded onto a precast ready gel 12% Tris-HCl. Proteins were separated electrophoretically and transferred to polyvinylidene difluoride (PVDF) membranes using the BioRad Electrophoretic Transfer Cell. For immunoblotting, membranes were blocked with 10% non-fat dried milk in TPBS for 2 h. The primary antibodies employed were rabbit polyclonal antibodies against cyclin D1, PCNA, cyclin E and p27. After washing, appropriate secondary antibody (anti-rabbit IgG-peroxidase conjugated) was applied for 1h. Blots were washed, incubated in commercial enhanced chemiluminescence reagents and exposed to chemiluminescence film. Quantification of the films was performed by a laser densitometer (Molecular Dynamics, CA, USA).

### 4.6. Statistical Analysis

The results were calculated as the means ± SD of four experimental observations in duplicate (four animals). Differences between groups were analyzed by an ANOVA following Snedecor F (α = 0.05). Students’ test was performed for statistical evaluation as follows: (a) all values against their control; (b) differences between two groups GD + TA versus TA.

## 5. Conclusions

We conclude that gadolinium chloride promotes and accelerates cell proliferation induced by thioacetamide. These facts are in accordance with the acceleration of liver regeneration observed in rats treated with gadolinium chloride and described by other authors. This proliferation marker indicates, as in the case of cyclins D1 and E, that the process of cell proliferation in post necrotic liver regeneration was anticipate in rats pretreated with the Kupffer cells inhibitor. However, the effects observed in the regeneration cannot be attributed exclusively to the changes seen in the proteins, as the complex liver regeneration process involves numerous cytokines whose signaling pathways are not completely understood at present. Blocking the function of Kupffer cells by gadolinium chloride apparently interrupted a step in the sequence of events leading to hepatotoxicity.
